# Patellofemoral Joint Replacement for Isolated Patellofemoral Osteoarthritis: Mid- to Long-Term Survivorship and Functional Outcomes

**DOI:** 10.3390/jpm16070345

**Published:** 2026-06-25

**Authors:** Fernando Diaz Dilernia, Mutaz Tageldein, Emad Anam, Aaron Campbell, Gavin Wood

**Affiliations:** 1 Division of Orthopaedic Surgery, Department of Surgery, Queen’s University, Kingston, ON K7L 3N6, Canada; aaron.campbell@kingstonhsc.ca; 2 Michael G. DeGroote School of Medicine, McMaster University, Hamilton, ON L8S 4L8, Canada; mutaz.mohamed@medportal.ca; 3 Orthopedic Department, Faculty of Medicine, King Abdulaziz University, Jeddah 21589, Saudi Arabia; eaanam@kau.edu.sa; 4 Division of Orthopaedics, Department of Surgery, McMaster University, Hamilton, ON L8S 4L8, Canada; orthowood@gmail.com

**Keywords:** patellofemoral arthritis, patellofemoral joint replacement, anterior knee pain, functional outcomes, survival rate

## Abstract

**Background/Objectives:** Patellofemoral joint (PFJ) replacement is a bone-preserving option for isolated patellofemoral osteoarthritis; however, reported survivorship and failure patterns remain variable. This study evaluated implant survivorship, functional outcomes, reoperations, and failure mechanisms following PFJ replacement using standard second-generation implant systems, with or without patellar resurfacing. **Methods:** We retrospectively reviewed a consecutive cohort of 39 patients (48 knees) who underwent PFJ replacement for isolated patellofemoral osteoarthritis between 2011 and 2021. Median age at surgery was 59 years, and median body mass index (BMI) was 31 kg/m^2^. Median follow-up for clinical and revision surveillance was 9 years (IQR 8–10). Functional outcomes were assessed using the Oxford Knee Score (OKS) and SF-12 Physical and Mental Component Scores (PCS and MCS). Implant survivorship was analyzed using Kaplan–Meier methodology, with conversion to total knee arthroplasty (TKA) as the endpoint. Statistical analyses were primarily descriptive and exploratory because only five TKA revisions occurred. **Results:** Median OKS improved from 19 (IQR 16–24) preoperatively to 36 (IQR 24–42) at the latest follow-up, with a median paired improvement of 17 points. SF-12 PCS improved from 25 to 47, and SF-12 MCS from 36 to 55. Eight knees (16.7%) underwent non-revision reoperation, and five knees (10.4%) underwent conversion to TKA. All TKA revisions were performed for the progression of tibiofemoral osteoarthritis. Kaplan–Meier survivorship free from TKA revision was 89.6% at 9 years (95% CI 76.8–95.5). No clear difference in TKA-free survivorship was detected between resurfaced and non-resurfaced knees. **Conclusions:** PFJ replacement demonstrated substantial functional improvement and mid- to long-term survivorship comparable to published registry ranges in a selected cohort with isolated patellofemoral osteoarthritis. TKA revision was uncommon and was attributable to the progression of tibiofemoral osteoarthritis. Because of the retrospective design, small cohort size, bilateral cases, and limited number of revision events, subgroup and risk-factor analyses should be interpreted as exploratory.

## 1. Introduction

Patellofemoral joint (PFJ) osteoarthritis is a common cause of anterior knee pain and has been reported in approximately 26% of men and 41% of women in population-based studies [1]. Isolated patellofemoral osteoarthritis is clinically distinct from tricompartmental knee osteoarthritis because symptoms are often localized to the anterior knee and are commonly exacerbated by stairs, rising from a seated position, squatting, kneeling, and activities requiring loaded knee flexion. The condition may arise from degenerative cartilage loss, trochlear dysplasia, chronic patellar maltracking, prior instability, post-traumatic change, or altered patellofemoral contact mechanics [2,3]. Initial management is typically non-operative and includes physiotherapy, activity modification, weight optimization, analgesic strategies, injections when appropriate, and treatment of contributing malalignment or instability [2,3]. Surgical intervention is generally reserved for patients with persistent symptoms despite conservative treatment and radiographic disease isolated to the patellofemoral compartment.

Surgical options for isolated patellofemoral osteoarthritis include arthroscopic procedures, realignment osteotomies, patellofemoral joint replacement, and total knee arthroplasty (TKA) [2,3,4,5]. PFJ replacement is intended to preserve the tibiofemoral compartments, cruciate ligaments, menisci, and tibiofemoral bone stock while resurfacing the diseased patellofemoral articulation. In appropriately selected patients, this bone- and ligament-preserving approach may offer advantages over TKA, including more native knee kinematics, faster early recovery, reduced perioperative morbidity, lower blood loss, shorter operative time, and preservation of future conversion options [5,6,7,8,9]. However, the procedure remains technically and biologically sensitive, and its durability depends heavily on patient selection, implant design, component positioning, patellar tracking, and the absence of clinically meaningful tibiofemoral disease.

Modern second-generation PFJ replacement systems were developed to address limitations of earlier implant designs. Contemporary onlay designs use a broader trochlear flange and improved trochlear geometry to optimize patellar engagement and reduce maltracking, edge loading, and instability [2,3]. Despite these advances, PFJ replacement remains associated with higher revision rates than TKA in registry and comparative studies [5,6,7,8,9,10]. The most common causes of failure include progression of tibiofemoral osteoarthritis, persistent pain, maltracking or instability, and less commonly loosening or component-related mechanical failure [4,5,10]. Therefore, the procedure is best understood as a joint-preserving option for selected patients rather than a direct durability equivalent to TKA.

Recent registry and systematic review data have clarified expected survivorship ranges, but institutional mid- to long-term data remain useful because outcomes vary by implant system, surgical technique, patellar resurfacing strategy, surgeon experience, and cohort selection [6,7,10]. In particular, there remains uncertainty regarding the contribution of patellar resurfacing, patient factors, and radiographic disease progression to revision risk. We hypothesized that patients with isolated PFJ osteoarthritis treated at our institution would demonstrate implant survival comparable to that reported in international registries. The primary objective of this study was to evaluate implant survivorship following PFJ replacement at our institution. Secondary objectives included assessing functional outcomes, identifying failure mechanisms, and exploring potential demographic and surgical predictors of revision.

## 2. Methods

This study was approved by the Biomedical Ethics Research Committee of Queen’s University.

We retrospectively reviewed a consecutive institutional cohort of 39 patients (48 knees) who underwent PFJ replacement between 2011 and 2021 (Table 1). Nine patients underwent bilateral PFJ replacement. Detailed screening logs were not available; therefore, the numbers of potentially eligible knees, excluded knees, unreachable patients, patients declining participation, or patients lost before PROM completion could not be reconstructed. Patients were eligible if they had symptomatic isolated patellofemoral osteoarthritis with anterior knee pain and clinically and radiographically preserved tibiofemoral compartments. Preoperative assessment included standard weight-bearing knee radiographs, including anteroposterior, lateral, and patellofemoral views. MRI was obtained selectively when the extent of tibiofemoral disease was uncertain. Exclusionary features included clinically meaningful tibiofemoral osteoarthritis, substantial tibiofemoral joint-space narrowing, inflammatory arthritis, major malalignment, or active instability requiring an additional procedure. Patients with tibiofemoral disease sufficient to justify primary TKA were not considered candidates for isolated PFJ replacement.

All procedures were performed by two high-volume academic arthroplasty surgeons using standard medial parapatellar or midvastus approaches. Standard cemented PFJ arthroplasty systems were used, predominantly Zimmer Biomet NexGen (Warsaw, IN, USA) and Smith & Nephew Journey implants (Memphis, TN, USA). Implant selection, surgical approach, and patellar resurfacing strategy were based on surgeon preference, implant availability, patient anatomy, patellofemoral pathology, and intraoperative findings (Table 2). Component positioning aimed to restore trochlear tracking and avoid overstuffing. Patellar tracking was assessed intraoperatively through range of motion. Lateral release was not routine and was used only selectively if tracking remained unacceptable after component positioning. Patellar resurfacing was considered when there was substantial patellar cartilage loss, severe patellar-side disease, or concern that leaving the patella unresurfaced would contribute to persistent anterior knee pain; this decision was not based on a predefined radiographic algorithm. Postoperatively, patients were permitted to weight-bear as tolerated and received routine thromboprophylaxis for 6 weeks. Immediate postoperative radiographs were reviewed and compared with the most recent follow-up radiographs. Progression of tibiofemoral osteoarthritis leading to conversion to TKA was determined from treating-surgeon documentation and review of follow-up radiographs demonstrating interval tibiofemoral degenerative progression. A standardized radiographic grading system was not applied retrospectively.

Patients were contacted for follow-up and provided informed consent for participation. Complete follow-up data were available for all included knees at the latest assessment. Follow-up duration was calculated from the date of index PFJ replacement to the latest clinical/revision surveillance follow-up or to revision to TKA. The reported 9-year median follow-up reflects clinical and revision surveillance. PROMs and radiographs were obtained at the latest available assessment when possible. The OKS was treated as a knee-specific outcome. SF-12 PCS and MCS are patient-level health-status measures; thus, analyses involving the SF-12 were interpreted with caution in bilateral cases. Questionnaires were completed electronically or in paper format and recorded in a secure institutional database.

Continuous variables were summarized as medians with interquartile ranges as distributions were non-normal on visual inspection and Shapiro–Wilk testing. Categorical variables were summarized as frequencies and percentages. Between-group comparisons for resurfaced versus non-resurfaced knees were performed using Mann–Whitney U tests for continuous variables and Fisher’s exact or chi-squared tests for categorical variables, as appropriate. Paired preoperative and postoperative PROMs were compared using Wilcoxon signed-rank tests. Median paired changes were reported with bootstrap 95% confidence intervals. Implant survivorship was analyzed using Kaplan–Meier methods, with conversion to TKA as the endpoint. Knees not revised to TKA were censored at the latest clinical/revision surveillance follow-up. Survival estimates were reported with 95% confidence intervals and numbers at risk. Resurfaced and non-resurfaced knees were compared using the log-rank test. Given the small number of TKA revision events, regression analyses were considered exploratory; no multivariable predictive model was used to support inference. The primary analyses were performed at the knee level. A sensitivity analysis of TKA survivorship was performed by restricting the cohort to one index knee per patient, defined as the first operated knee. Statistical significance was defined as *p* < 0.05. All analyses were performed using SPSS version 27 (SPSS Inc., Chicago, IL, USA).

## 3. Results

### 3.1. Functional Outcomes

**Figure 1:**
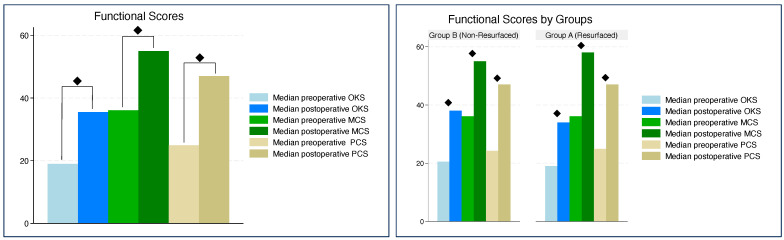
Comparison of preoperative and latest postoperative OKS, SF-12, PCS, and SF-12 MCS following PFJ replacement. OKS: Oxford Knee Score; SF-12: 12-item Short Form Survey; PCS: Physical Component Score; MCS: Mental Component Score.

Patient-reported outcome measures improved substantially following surgery (Figure 1, Table 3). Median OKS improved from 19 (IQR 16–24) preoperatively to 36 (IQR 24–42) postoperatively at the latest follow-up, with a median paired improvement of 17 points (bootstrap 95% CI 8.0–20.5; *p* < 0.001). Median SF-12 MCS improved from 36.1 to 55.0, with a median paired improvement of 16.5 points (bootstrap 95% CI 15.5–22.0; *p* < 0.001). Median SF-12 PCS improved from 24.9 to 47.0, with a median paired improvement of 13.5 points (bootstrap 95% CI 8.1–22.6; *p* < 0.001). No significant differences in postoperative scores or paired change scores were observed between resurfacing groups. Median follow-up duration was longer in the resurfaced group.

### 3.2. Complications and Reoperations

No intraoperative complications were recorded. Postoperative complications included anterior knee pain in 6 knees (12.5%), stiffness in 2 knees (4.2%), and mechanical or meniscal-type symptoms in 4 knees (8.3%) (Table 4). Eight knees (16.7%) underwent non-revision reoperation, including secondary patellar resurfacing, manipulation under anesthesia, or arthroscopy as clinically indicated. Non-revision reoperations and TKA revisions were analyzed separately.

### 3.3. Implant Failure and Survivorship

**Figure 2:**
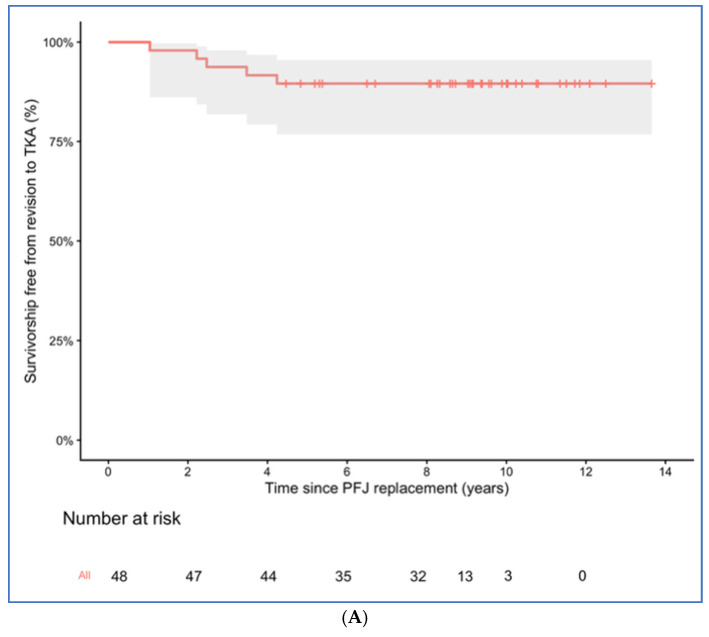

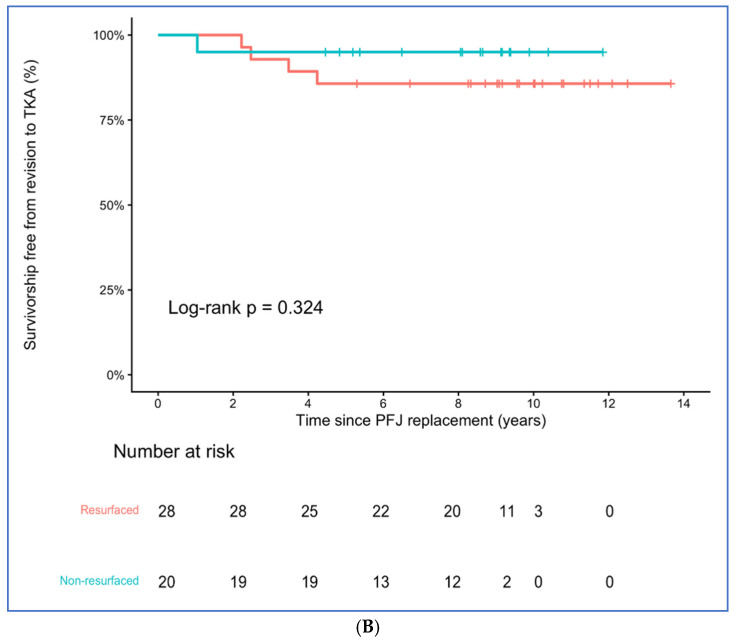
(**A**) Kaplan–Meier survivorship free from conversion to total knee arthroplasty. Shaded bands represent 95% confidence intervals. Censored observations indicate knees not revised to TKA at latest clinical/revision surveillance follow-up. (**B**) Kaplan–Meier implant survivorship free from conversion to total knee arthroplasty by patellar resurfacing status. Censored observations indicate knees not revised to TKA at latest clinical/revision surveillance follow-up. Resurfaced and non-resurfaced knees were compared using the log-rank test.

Five knees (10.4%) underwent revision to TKA. All revisions were performed for the progression of tibiofemoral osteoarthritis. Kaplan–Meier survivorship free from revision to TKA was 89.6% at 9 years (95% CI 76.8–95.5) (Figure 2). Numbers at risk at 0, 2, 4, 6, 8, 9, and 10 years were 48, 47, 44, 35, 32, 24, and 13 knees, respectively. No statistically significant difference in TKA-free survivorship was observed between resurfaced and non-resurfaced knees by log-rank testing (*p* = 0.324). In the index-knee sensitivity analysis, 9-year TKA-free survivorship was 87.2% (95% CI 71.9–94.5), with no significant difference between resurfacing groups (Table S1).

### 3.4. Risk Factor Analysis

Because only five knees underwent conversion to TKA, the study was underpowered for stable multivariable prediction modeling. Exploratory univariable logistic regression did not identify statistically significant associations between conversion to TKA and age, sex, BMI, obesity status, prior surgery, or patellar resurfacing (Table S2). These analyses should be interpreted as hypothesis-generating only and were not used to define independent predictors of revision.

## 4. Discussion

This study demonstrates that PFJ replacement can provide substantial functional improvement and acceptable mid- to long-term survivorship in selected patients with isolated patellofemoral osteoarthritis. The observed 10.4% conversion rate to TKA and 9-year Kaplan–Meier survivorship of 89.6% are broadly consistent with contemporary registry and systematic review data. Recent pooled analyses have reported approximately 90% survivorship at 5 years and low- to mid-80% survivorship at 10 years, with survivorship declining further with longer follow-up [11]. In the present cohort, all revisions to TKA were performed for progression of tibiofemoral osteoarthritis rather than unexplained anterior knee pain, aseptic loosening, or implant breakage. This failure pattern is clinically important because it suggests that, in this cohort, implant-related mechanical failure was uncommon and that the dominant long-term threat to PFJ replacement durability was progression of disease in the retained tibiofemoral compartments.

The finding that tibiofemoral osteoarthritis progression accounted for all TKA revisions reinforces the central role of preoperative selection. PFJ replacement preserves the tibiofemoral compartments, which is a major theoretical advantage in younger or more active patients with truly isolated disease. The same feature also creates the main biological vulnerability of the operation: subtle or under-recognized tibiofemoral degeneration, meniscal pathology, malalignment, or inflammatory disease may progress after surgery and ultimately require conversion to TKA. This supports careful preoperative assessment with weight-bearing radiographs, patellofemoral views, and selective MRI when tibiofemoral cartilage status is uncertain. It also supports explicit patient counseling that PFJ replacement does not prevent later tibiofemoral disease progression, even when index surgery is technically successful.

No clear association was detected between patellar resurfacing and TKA revision, postoperative PROMs, or paired PROM improvement. However, this comparison should be interpreted cautiously. Patellar resurfacing was not randomized and was determined by surgeon preference, patellar cartilage status, patellar morphology, and intraoperative judgment. Follow-up duration also differed between groups, and implant distribution was not identical. Therefore, the resurfacing analysis is descriptive rather than definitive and should not be interpreted as evidence that patellar resurfacing is unimportant. The two secondary patellar resurfacing procedures in the non-resurfaced group also suggest that residual patellar-side disease may remain clinically relevant in selected patients, although the present study is too small and non-randomized to determine whether routine patellar resurfacing improves outcomes.

Functional outcomes in our cohort improved substantially and were comparable to those reported in contemporary PFJ arthroplasty literature. Although MCID thresholds specific to PFJ replacement are not well established, the observed median OKS improvement of 17 points and SF-12 PCS improvement of 13.5 points exceed reported MCID estimates for OKS and SF-12 after TKA, supporting the clinical relevance of the observed PROM improvements [12]. These findings align with recent reviews and comparative studies suggesting that modern PFJ replacement can provide meaningful pain relief and functional recovery in appropriately selected patients [3,5,8,13]. Contemporary comparative evidence generally frames PFJ replacement and TKA as a tradeoff rather than a simple superiority question: PFJ replacement may provide more tissue preservation, lower early resource use, and favorable early recovery, whereas TKA generally demonstrates lower revision risk over longer follow-up [5,6,7,8,9]. Therefore, the clinical value of PFJ replacement depends on selecting patients in whom the benefits of compartment preservation outweigh the increased probability of later revision.

Recent comparative studies have further clarified this tradeoff. Large database and registry analyses have reported that PFJ replacement may be associated with fewer early medical complications, reduced length of stay, and lower short-term cost compared with TKA, but with a higher likelihood of revision over time [5,8]. A recent meta-analysis similarly suggested that modern PFJ replacement may reduce operative time and blood loss and may provide favorable early functional recovery, while longer-term durability remains the principal concern [9]. These findings are consistent with the present cohort, in which functional improvement was substantial and non-revision complications were limited, but conversion to TKA remained the defining survivorship endpoint.

This study has several limitations. Its retrospective design and absence of a control group limit causal inference. The cohort was small, and only five TKA revisions occurred, limiting statistical power and preventing stable multivariable prediction modeling. Analyses were primarily performed at the knee level, with 48 knees coming from 39 patients; this may introduce within-patient correlation, particularly for bilateral cases. An index-knee sensitivity analysis was therefore performed, but this does not fully eliminate the limitations of the original design. Implant selection, surgical approach, and patellar resurfacing were based on surgeon preference and intraoperative judgement rather than a standardized treatment algorithm, which may introduce selection bias. Our data did not include sufficient demographic information, detailed comorbidity data, or perioperative variables that could contribute to the development of complications. Additionally, longer follow-up may reveal additional revisions; therefore, reported survivorship should be interpreted as a best-case estimate. Finally, it is essential to note that surgical experience and familiarity with these implants may be crucial for postoperative outcomes. High-volume arthroplasty surgeons performed all procedures, and outcomes may not be directly generalizable to lower-volume settings.

Despite these limitations, this study provides institutional mid- to long-term data regarding survivorship, functional improvement, and failure patterns following PFJ replacement. These findings reinforce the importance of appropriate patient selection and counseling regarding the potential progression of tibiofemoral disease. Future studies with larger cohorts, standardized radiographic grading, longer uniform follow-up, and analytic methods that account for bilateral cases are needed to better define which patients benefit most from PFJ replacement and to clarify the role of patellar resurfacing. Future work should also evaluate whether standardized radiographic grading, objective assessment of patellofemoral tracking, improved patient-specific planning, and emerging technologies such as robotic-assisted PFJ replacement can improve reproducibility of component positioning and reduce failure related to maltracking, overstuffing, and progression of adjacent-compartment disease [13].

## 5. Conclusions

At a median clinical/revision surveillance follow-up of approximately nine years, PFJ replacement was associated with substantial functional improvement and survivorship free from TKA revision in selected patients with isolated patellofemoral osteoarthritis. TKA revision occurred in a minority of knees and was attributable to progression of tibiofemoral osteoarthritis. No clear predictors of revision were identified, but the study was underpowered for predictive modeling. These findings support PFJ replacement as a reasonable joint-preserving option for carefully selected patients, while emphasizing the importance of counseling about subsequent tibiofemoral disease progression and possible conversion to TKA.

## Figures and Tables

**Table 1:** Demographic and Clinical Characteristics.

Variable	Overall	Group A: Resurfaced	Group B: Non-Resurfaced	*p* Value
Patients (knees), n	39 (48)	22 (28)	17 (20)	N/A
Age at surgery, years	59 (IQR, 53–70)	55 (IQR, 52–71)	62 (IQR, 56–70)	0.34
Sex, women, n (%)	28/39 (71.8)	14/22 (63.6)	14/17 (82.4)	0.20
Body mass index, kg/m^2^	31 (IQR, 25–32)	31 (IQR, 25–33)	31 (IQR, 26–32)	0.77
Obesity classification, n (%)				0.515
Class I	21 (72.4)	10 (62.5)	11 (84.6)	
Class II	1 (3.5)	1 (6.3)	0 (0.0)	
Class III	7 (24.1)	5 (31.2)	2 (15.4)	
Comorbidities, n (%)				
Hypertension	14 (29.2)	7 (25.0)	7 (35.0)	0.45
Current/former smoking	5 (10.4)	4 (14.3)	1 (5.0)	0.30
Diabetes mellitus	4 (8.3)	1 (3.6)	3 (15.0)	0.16
Diagnosis, knees, n (%)				
Isolated patellofemoral osteoarthritis	48 (100)	28 (100)	20 (100)	N/A
Prior ipsilateral knee surgery, knees, n (%)	10 (20.8)	6 (21.4)	4 (20.0)	0.90
Arthroscopy	8/10 (80.0)	5/6 (83.3)	3/4 (75.0)	
Tibial tubercle osteotomy	2/10 (20.0)	1/6 (16.7)	1/4 (25.0)	
Follow-up for clinical/revision surveillance, years	9 (IQR, 8–10)	10 (IQR, 8.75–10.25)	8 (IQR, 5–9)	0.002

Values are median (IQR) unless otherwise stated. Categorical values are n (%) or n/N (%). Primary analyses are knee-level unless otherwise specified. Sex and patient count are patient-level. N/A: Not applicable.

**Table 2:** Operative Characteristics.

Variable	Overall (n = 48)	Group A: Resurfaced (n = 28)	Group B: Non-Resurfaced (n = 20)	*p* Value
Surgical approach, n (%)				0.28
Medial parapatellar	26 (54.2)	17 (60.7)	9 (45.0)	
Midvastus	22 (45.8)	11 (39.3)	11 (55.0)	
Implant system, n (%)				0.07
Zimmer Biomet NexGen	40 (83.3)	21 (75.0)	19 (95.0)	
Smith & Nephew Journey	8 (16.7)	7 (25.0)	1 (5.0)	
Femoral component size, n (%)				0.43
Zimmer Biomet NexGen size 2	6 (12.5)	2 (7.1)	4 (20.0)	
Zimmer Biomet NexGen size 3	13 (27.1)	9 (32.1)	4 (20.0)	
Zimmer Biomet NexGen size 4	16 (33.3)	8 (28.6)	8 (40.0)	
Zimmer Biomet NexGen size 5	5 (10.4)	2 (7.1)	3 (15.0)	
Smith & Nephew Journey extra-small	3 (6.3)	2 (7.1)	1 (5.0)	
Smith & Nephew Journey small	2 (4.2)	2 (7.1)	0 (0.0)	
Smith & Nephew Journey medium	2 (4.2)	2 (7.1)	0 (0.0)	
Smith & Nephew Journey large	1 (2.1)	1 (3.6)	0 (0.0)	
Patellar component details				
Median patellar component size, mm	N/A	29 (IQR, 29–32)	N/A	N/A
Median patellar component thickness, mm	N/A	8.5 (IQR, 8–8.5)	N/A	N/A
NexGen all-poly size, mm	N/A	29 (IQR, 29–32)	N/A	N/A
NexGen all-poly thickness, mm	N/A	8 (IQR, 8–8.5)	N/A	N/A
Genesis II biconvex size, mm	N/A	29 (IQR, 29–30.5)	N/A	N/A
Genesis II biconvex thickness, mm	N/A	8.5 (IQR, 8.5–8.75)	N/A	N/A

Values are n (%) or median (IQR). N/A: Not applicable.

**Table 3:** Preoperative and Postoperative Functional Outcomes.

Statistic	Overall	Group A: Resurfaced	Group B: Non-Resurfaced	*p* Value: Group A vs. B
Oxford Knee Score	n = 48	n = 28	n = 20	
Preoperative score, median (IQR)	19 (IQR, 16–24)	19 (IQR, 16–20.3)	21 (IQR, 18.3–24)	0.15
Latest postoperative score, median (IQR)	36 (IQR, 24–42)	34 (IQR, 24–42)	38 (IQR, 24–42)	0.51
Paired change, median (bootstrap 95% CI)	17 (95% CI, 8.0–20.5)	18.5 (95% CI, 5.0–24.0)	16 (95% CI, 10.5–19.0)	0.72
Within-group pre/post *p* value	<0.001	<0.001	<0.001	N/A
SF-12 MCS	n = 39	n = 22	n = 17	
Preoperative score, median (IQR)	36.1 (IQR, 31.7–41.6)	36.1 (IQR, 31.7–41.6)	36.1 (IQR, 31.7–41.6)	0.84
Latest postoperative score, median (IQR)	55 (IQR, 52–58.1)	58 (IQR, 50.3–58.1)	55 (IQR, 52–58)	0.70
Paired change, median (bootstrap 95% CI)	16.5 (95% CI, 15.5–22.0)	18.9 (95% CI, 13.7–22.7)	16.5 (95% CI, 13.4–22.0)	0.80
Within-group pre/post *p* value	<0.001	<0.001	<0.001	N/A
SF-12 PCS	n = 39	n = 22	n = 17	
Preoperative score, median (IQR)	24.9 (IQR, 24.2–33.5)	24.9 (IQR, 24.2–33.5)	24.2 (IQR, 24.2–33.5)	0.48
Latest postoperative score, median (IQR)	47 (IQR, 25.3–47.5)	47 (IQR, 25.3–47.5)	47 (IQR, 26.8–47.5)	0.64
Paired change, median (bootstrap 95% CI)	13.5 (95% CI, 8.1–22.6)	13.5 (95% CI, 3.4–22.7)	13.5 (95% CI, 8.1–23.3)	0.50
Within-group pre/post *p* value	<0.001	0.003	0.001	N/A

N/A: Not applicable.

**Table 4:** Postoperative Complications, Non-revision Reoperations, and TKA Revision.

Outcome	Overall (n = 48)	Group A: Resurfaced (n = 28)	Group B: Non-Resurfaced (n = 20)	Treatment/Endpoint	*p* Value
Postoperative symptoms/complications, n (%)					
Anterior knee pain	6 (12.5)	4 (14.3)	2 (10.0)	Physiotherapy or further treatment as clinically indicated	0.66
Stiffness	2 (4.2)	1 (3.6)	1 (5.0)	Manipulation under anesthesia	0.81
Mechanical/meniscal-type symptoms	4 (8.3)	4 (14.3)	0 (0.0)	Arthroscopy	0.14
Non-revision reoperations, n (%)					
Any non-revision reoperation	8 (16.7)	5 (17.9)	3 (15.0)	MUA, arthroscopy, or secondary patellar resurfacing	0.79
Manipulation under anesthesia	2 (4.2)	1 (3.6)	1 (5.0)	For stiffness	N/A
Arthroscopy	4 (8.3)	4 (14.3)	0 (0.0)	For mechanical/meniscal-type symptoms	N/A
Secondary patellar resurfacing	2 (4.2)	0 (0.0)	2 (10.0)	For persistent anterior knee pain after index non-resurfacing	N/A
TKA revision, n (%)					
Conversion to TKA	5 (10.4)	4 (14.3)	1 (5.0)	Endpoint for Kaplan–Meier survivorship	0.30
Indication: tibiofemoral OA progression	5/5 (100)	4/4 (100)	1/1 (100)	All TKA revisions	N/A

OA: osteoarthritis; MUA: manipulation under anesthesia; TKA: total knee arthroplasty. Symptom and complication categories were not treated as mutually exclusive; knees could contribute to more than one symptom category when clinically applicable. N/A: Not applicable.

## Data Availability

The data presented in this study are available from the corresponding author upon reasonable request and subject to institutional ethics approval.

## Supplementary Materials

The following supporting information can be downloaded at: https://www.mdpi.com/article/10.3390/jpm16070345/s1, Table S1: Index-knee sensitivity analysis for TKA-free survivorship; Table S2: Exploratory Univariable Logistic Regression for TKA revision.
