# Supplementary material for: Patellofemoral Joint Replacement for Isolated Patellofemoral Osteoarthritis: Mid- to Long-Term Survivorship and Functional Outcomes
Source: J Pers Med. 2026 Jun 25;16(7):345. doi: 10.3390/jpm16070345 (PMC13413508; doi:10.3390/jpm16070345)
Supplement: Supplementary file 1 [file jpm-16-00345-s001.zip › jpm-4318164-supplementary.pdf]

Supplementary Table S1. Index-knee sensitivity analysis for TKA-free survivorship.

Strata	Time (years)	Number at risk	Events	Survivorship (%)	95% Confidence interval
Overall	5	32	5	87.2	71.9-94.5
Overall	9	20	0	87.2	71.9-94.5
Overall	10	11	0	87.2	71.9-94.5
Resurfaced	5	18	4	81.8	58.5-92.8
Resurfaced	9	13	0	81.8	58.5-92.8
Resurfaced	10	9	0	81.8	58.5-92.8
Non-resurfaced	5	14	1	94.1	65-99.1
Non-resurfaced	9	7	0	94.1	65-99.1
Non-resurfaced	10	2	0	94.1	65-99.1

Supplementary Table S2. Exploratory Univariable Logistic Regression for TKA revision

Variable	Odds ratio	Standard error	P value	95% confidence interval
Age at surgery	0.93	0.04	0.101	0.86 to 1.01
Men	0.37	0.42	0.381	0.04 to 3.46
Body mass index	0.90	0.06	0.127	0.79 to 1.03
Obesity status	0.26	0.21	0.094	0.05 to 1.26
Previous ipsilateral surgery	3.64	3.17	0.137	0.66 to 20.01
Patella resurfaced	0.94	0.78	0.945	0.19 to 4.78
